# Pim Kinases Promote Migration and Metastatic Growth of Prostate Cancer Xenografts

**DOI:** 10.1371/journal.pone.0130340

**Published:** 2015-06-15

**Authors:** Niina M. Santio, Sini K. Eerola, Ilkka Paatero, Jari Yli-Kauhaluoma, Fabrice Anizon, Pascale Moreau, Johanna Tuomela, Pirkko Härkönen, Päivi J. Koskinen

**Affiliations:** 1 Section of Genetics and Physiology, Department of Biology, University of Turku, 20500 Turku, Finland; 2 Drug Research Doctoral Programme, University of Turku, 20520 Turku, Finland; 3 Institute of Biomedicine, Department of Medical Biochemistry and Genetics, University of Turku, 20520 Turku, Finland; 4 Division of Pharmaceutical Chemistry and Technology, Faculty of Pharmacy, University of Helsinki, 00014 Helsinki, Finland; 5 Institut de Chimie de Clermont-Ferrand, Université Clermont Auvergne, Université Blaise Pascal, 63000 Clermont-Ferrand, France; 6 Centre National de la Recherche Scientifique, 63178 Aubiere, France; 7 Institute of Biomedicine, Department of Cell Biology and Anatomy, University of Turku, 20520 Turku, Finland; 8 Pharmatest Services Ltd, 20520 Turku, Finland; University of Florida, UNITED STATES

## Abstract

**Background and methods:**

Pim family proteins are oncogenic kinases implicated in several types of cancer and involved in regulation of cell proliferation, survival as well as motility. Here we have investigated the ability of Pim kinases to promote metastatic growth of prostate cancer cells in two xenograft models for human prostate cancer. We have also evaluated the efficacy of Pim-selective inhibitors to antagonize these effects.

**Results:**

We show here that tumorigenic growth of both subcutaneously and orthotopically inoculated prostate cancer xenografts is enhanced by stable overexpression of either Pim-1 or Pim-3. Moreover, Pim-overexpressing orthotopic prostate tumors are highly invasive and able to migrate not only to the nearby prostate-draining lymph nodes, but also into the lungs to form metastases. When the xenografted mice are daily treated with the Pim-selective inhibitor DHPCC-9, both the volumes as well as the metastatic capacity of the tumors are drastically decreased. Interestingly, the Pim-promoted metastatic growth of the orthotopic xenografts is associated with enhanced angiogenesis and lymphangiogenesis. Furthermore, forced Pim expression also increases phosphorylation of the CXCR4 chemokine receptor, which may enable the tumor cells to migrate towards tissues such as the lungs that express the CXCL12 chemokine ligand.

**Conclusions:**

Our results indicate that Pim overexpression enhances the invasive properties of prostate cancer cells *in vivo*. These effects can be reduced by the Pim-selective inhibitor DHPCC-9, which can reach tumor tissues without serious side effects. Thus, Pim-targeting therapies with DHPCC-9-like compounds may help to prevent progression of local prostate carcinomas to fatally metastatic malignancies.

## Introduction

The *pim* family genes were first identified as proviral integration sites for Moloney murine leukemia virus [1], but have later been shown to be involved in development of human lymphoid malignancies as well as solid tumors [2]. The proteins encoded by the three *pim* family genes are serine/threonine-specific kinases that have been shown to promote tumorigenesis by increasing both proliferation and survival of cells [2,3]. More recently, we and others have also implicated them in the regulation of migration and invasion of adherent cancer cells [4–6], while results from clinical studies show association of abnormally high levels of Pim kinases with more malignant cancers of epithelial origin [7–9].

Because of their emerging roles in cancer development, Pim kinases have become highly attractive as therapeutic targets [10–12]. There are also physiological and structural reasons to justify Pim kinases as drug targets. First, inactivation of Pim kinases is not expected to cause serious side effects, since mice deficient for all three Pim family members are viable [13]. Secondly, unique structural features within the hinge region connecting the N- and C-terminal lobes around the ATP-binding pocket render the Pim kinases constitutively active and enable design of highly selective inhibitors [14]. We have recently identified potent and selective Pim kinase inhibitors within two structurally unrelated groups of compounds, tetracyclic pyrrolocarbazoles [15] and tricyclic benzo[*cd*]azulenes [16]. We have also functionally validated them in both *in vitro* and cell-based assays [6, 17].

Tumor xenografts provide excellent physiological settings for preclinical proof-of-concept studies, both to identify therapeutic targets and to evaluate *in vivo* efficacy of compounds targeting them. Subcutaneous inoculation of PC-3 prostate cancer cells overexpressing either Pim-1 or Pim-2 into immunodeficient mice has previously been shown to result in larger tumors [18], but comparable data on Pim-3 has been lacking as also direct evidence for the ability of Pim kinases to contribute to formation of metastases. Yet information from cell-based motility assays as well as clinical data connect upregulation of Pim kinases to cancer cell migration, invasion and more malignant behaviour [4–9]. In addition, Pim-1 has been shown to regulate the CXCR4/CXCL12 chemokine pathway, which plays an important role in migration and invasion of both leukemic [4, 19] and prostate cancer cells [20–23].

In this study, we have assessed the effects of Pim kinases and their inhibitors using both subcutaneous and orthotopic mouse xenograft models for human prostate cancer. We demonstrate that overexpressed Pim-1 or Pim-3 kinases promote not only growth of PC-3 cell-derived xenografts, but also metastatic properties of orthotopically induced tumors, and that Pim-inhibitory compounds can prevent these effects. We also show that the Pim-promoted metastatic growth is associated with increased angiogenesis, lymphangiogenesis and CXCR4 phosphorylation.

## Results

### Pim-3 kinase enhances growth and metastatic properties of prostate cancer xenografts

**Fig 1:**
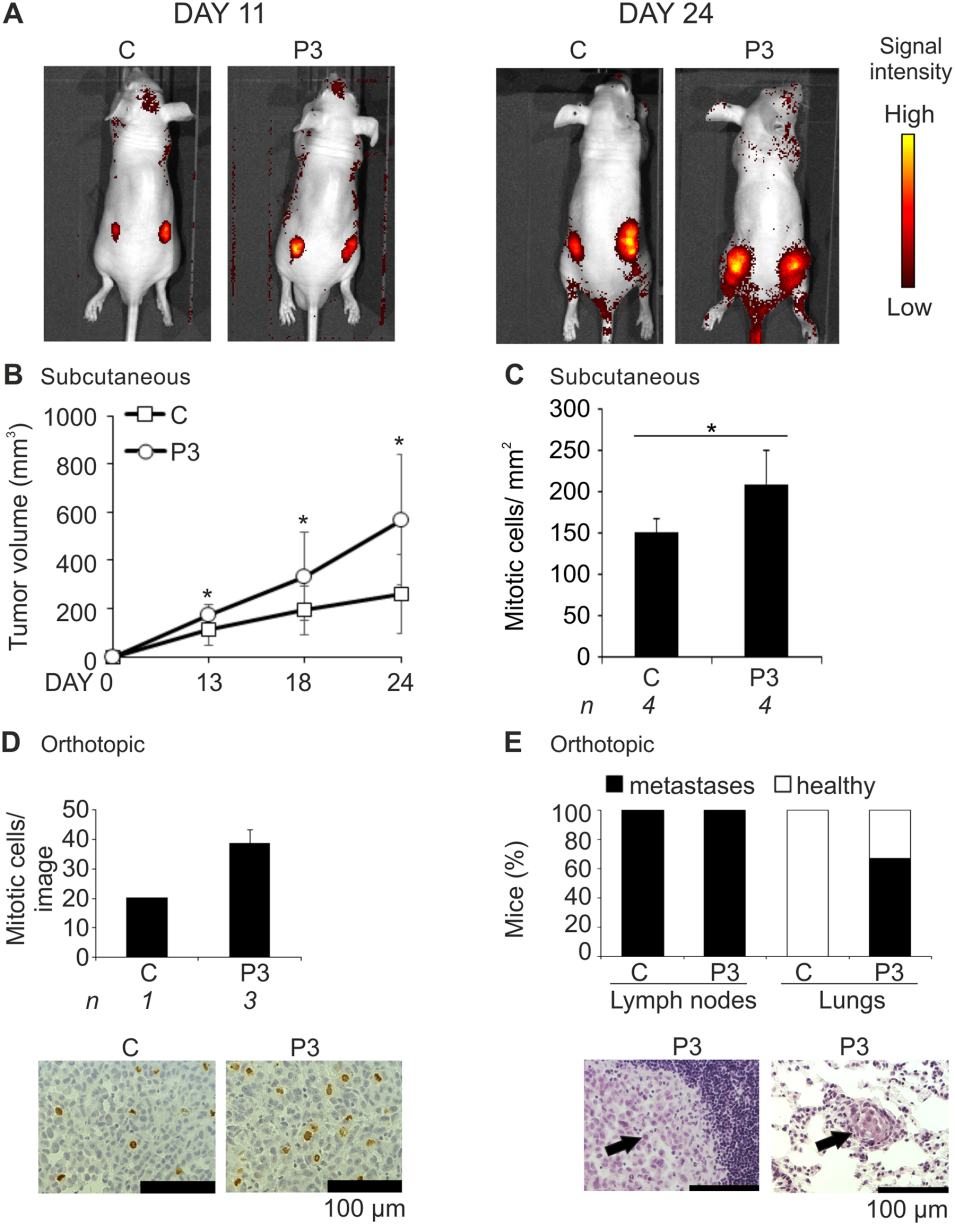
Pim-3 overexpression promotes metastatic growth of prostate tumor xenografts. PC-3-derived cell lines that had been stably transfected with an empty vector (C) or a vector expressing Pim-3 (P3) were subcutaneously or orthotopically injected into athymic nude mice. Tumors and isolated tissues were stained with Hematoxylin and Eosin for visualization of their structure. Additional stainings were carried out with anti-phospho-histone H3 antibody to visualize the number of mitotic cells. In the subcutaneous experiments, tumor formation was followed by fluorescence imaging of Tomato expression (A) and approximate tumor sizes were measured by palpation at different time-points (B). After 24 days, mice were sacrificed and their tumors and tissues were collected. Shown are average values from all fully imaged tumor sections from indicated numbers (*n*) of mice after staining of mitotic cells (C). In the orthotopic experiments, the stable PC-3 cells were allowed to grow in the prostates for three weeks. Thereafter mitotic cells (brown) were analysed from sample images (D), while metastases (indicated by arrows) were counted from prostate-draining lymph nodes and lungs (E).

To investigate the ability of Pim-3 to promote tumor growth and metastasis under *in vivo* conditions, we established a stable PC-3/Pim-3 prostate cancer cell line expressing human Pim-3 together with Tomato as a fluorescent follow-up marker. In order to evaluate the tumorigenic potential of the PC-3/Pim-3 cell line as compared to the mock-transfected PC-3 control cell line, cells were subcutaneously inoculated into athymic nude male mice. During the follow-up period of up to 24 days, tumor volumes were measured both with a caliper and by fluorescent imaging of Tomato expression. After sacrifice, tumors and tissue samples were excised for fluoro- and morphometric analyses. These revealed that the Pim-3-overexpressing xenografts had grown significantly faster than the mock-transfected cells, even though tumors had remained local without any signs of metastases (Fig 1A and 1B). The manually measured tumor volumes correlated with the areas determined by fluorescent imaging (S1 Fig). To further analyse the growth properties of these tumors, mitotic cells were stained from paraffin-embedded tissues samples. Interestingly, the proportion of mitotic cells was clearly higher in the Pim-3-overexpressing tumor tissues than in the controls (Fig 1C and S1 Fig). The differences in the tumor volumes could also be detected from the whole tumor scans used for analysis of the mitotic cells (S1 Fig). Simultaneously to the subcutaneous experiments, cells were cultured for three weeks without antibiotic selection to confirm the stability of Pim-3 overexpression (S1 Fig).

Since the subcutaneous xenografts had not invaded into the body, we continued our studies with an orthotopic prostate cancer model, where the tumor microenvironment was expected to be more favorable towards metastatic growth [24–26]. In the first pilot set of experiments, control or Pim-3-overexpressing cells were orthotopically inoculated into the prostates of nude male mice. Tumor growth was followed during a three-week period, after which the animals were sacrificed and tumors along with selected organs were collected. In this study, no major differences in tumor volumes were detected. However, analysis of paraffin-embedded tissue samples not only revealed the higher mitotic potential of the Pim-3-overexpressing cells, but also their ability to invade into the lungs (Fig 1D and 1E). By contrast, the milder metastatic behaviour of the mock-transfected control cells confirmed our previous observations on the ability of parental PC-3 cells to invade into prostate-draining lymph nodes, but rarely to more distant organs [25–26].

### Pim inhibition is tolerated by zebrafish embryos and adult mice

The promising results with invasive Pim-3-overexpressing orthotopic tumors prompted us to perform another set of experiments, where we also tested the effects of Pim inhibition by the tetracyclic pyrrolocarbazole DHPCC-9 [6] and the tricyclic benzo[*cd*]azulene BA-1a [17]. For comparison, we also established another stable PC-3 cell line overexpressing human Pim-1.

Prior to animal experimentation, the efficacy and toxicity of the Pim-selective inhibitors were tested in cell-based assays and *in vivo*. Both inhibitors efficiently antagonized the pro-migratory effects of Pim-1 and Pim-3, and decreased migration of all stable cell lines to a similar extent (S2 Fig). Within the 24 h follow-up period of the wound healing assay, cell viability was only slightly affected, while both inhibitors dramatically reduced it in all cell lines by a later 72 h time-point. When the *in vivo* safety of the inhibitors was analysed with zebrafish embryos within their aquatic environment, both DHPCC-9 and BA-1a were well tolerated, while our cytotoxic control compound BA-2c [17] led to massive developmental problems and death (S3 Fig and S1 Table). However, slightly curved tails and enlarged pericardiac sacs were observed in embryos treated with 10 μM DHPCC-9 (S3 Fig), suggesting that proper Pim activity is needed for normal embryonal development. Yet these data did not allow for reliable conclusions on the safety of the inhibitors in adult organisms.

Additional safety tests were then carried out with adult mice. However, with the high concentrations needed for these tests, only DHPCC-9 could be suspended in DMSO, while BA-1a was soluble only in N,N-dimethylacetamide (DMA).

During an initial ten-day follow-up period, DHPCC-9 treatments (50 mg/kg) caused no major changes in mouse behavior, injection area or body weight (S4 Fig). By contrast, DMA-based treatments (25 mg/kg) caused restless behavior and slightly decreased body weight. In addition, DMA seemed to induce scar tissue formation in the injection area. Thereafter, safety testing was continued with smaller amounts of DMA (10–20 mg/kg), which did not cause any visible changes in the injection area, mouse behavior or weight gain during the 17-day follow-up (S4 Fig). Based on these results, 50 mg/kg of DHPCC-9 in 20 μl of DMSO and 20 mg/kg of BA-1a in 10 μl of DMA were decided to be used daily to test their effects on orthotopic Pim-3-overexpressing prostate xenografts.

### Pim inhibition reduces Pim-dependent metastatic growth of orthotopic prostate xenografts

In the second set of orthotopic experiments, mock-transfected PC-3 cells or cells stably overexpressing Pim-1 or Pim-3 were orthotopically inoculated into the prostates of nude male mice. Mice with Pim-3-overexpressing xenografts were randomized into 4 groups and administered with daily dosages of the inhibitors or equal volumes of DMSO or DMA as controls. Mouse behavior and weight gain was followed during the experiment, but no major inhibitor-related changes were detected (S5 Fig). After sacrifice, tumors were excised and mice without tumors were excluded from further analyses (S2 Table). To confirm the stability of the cell lines, *ex vivo* scanning was performed to detect Tomato signals in tumors (S6 Fig), while immunohistochemistry was used to visualize the xenografted tumor cells expressing Pim proteins from V5-tagged constructs (S7 Fig).

**Fig 2:**
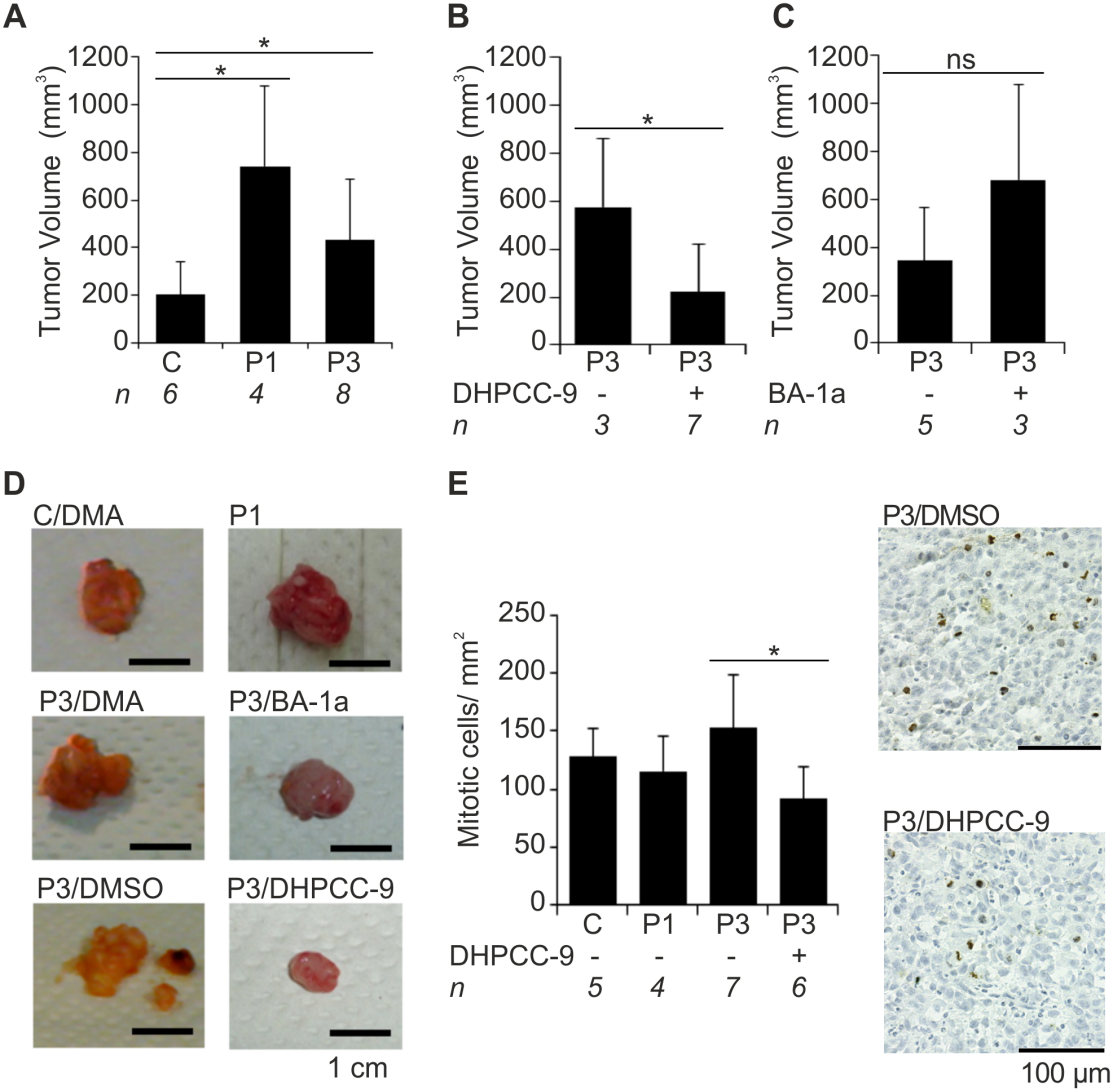
Pim overexpression increases and Pim inhibition by DHPCC-9 decreases growth of orthotopic prostate xenografts. PC-3-derived stably transfected cells (Mock = C, Pim-1 = P1, Pim-3 = P3) were orthotopically inoculated into the prostates of athymic nude mice. Mice were treated daily with either DMSO or DMA (control treatments) or with Pim inhibitors (50 mg/kg of DHPCC9 in DMSO or 20 mg/kg of BA-1a in DMA). After three weeks of treatments, mice were sacrificed and their tumor volumes were measured. First the volumes were compared between indicated numbers (*n*) of mice without inhibitor treatments (A). Then the volumes of tumors derived from inhibitor-treated animals were compared to tumors from animals with appropriate control treatments DMSO (B) or DMA (C). Before tumor fixation, representative images were taken (D). Later on paraffin-embedded tumor sections were stained with anti-phospho-histone H3 antibody to visualize mitotic cells (brown). Shown are average values combined from all fully imaged tumor tissue sections from DHPCC-9-treated and DMSO- or DMA-treated control groups as well as representative images from Pim-3-overexpressing tumors (E).

When tumor volumes were calculated, Pim-overexpressing tumors were again significantly larger than those formed by mock-transfected cells (Fig 2A and 2D). However, the Pim-1 xenografts could not be directly compared to others, since mice carrying them had not obtained any chemical treatments. DHPCC-9 treatment significantly decreased the volume of Pim-3-overexpressing tumors, suggesting that this compound had been able to reach the tumor tissue and inhibit Pim-3 activity there (Fig 2B and 2D). By contrast, BA-1a did not show any efficacy in terms of reducing tumor volume (Fig 2C and 2D). Apparently this compound had not reached its target tissue, as also suggested by the presence of a yellow precipitate around the injection site.

The mitotic rates were also measured from tissue sections derived from the orthotopic tumors. While in the subcutaneous experiments and in the first set of orthotopic experiments, there had been clearly more mitotic cells in tumors formed by Pim-3-overexpressing cells as compared to mock-transfected cells (Fig 1C and 1D), in the second orthotopic set the differences were smaller (Fig 2E). However, treatment with DHPCC-9 had decreased the number of mitotic cells in Pim-3 xenografts (Fig 2E).

**Fig 3:**
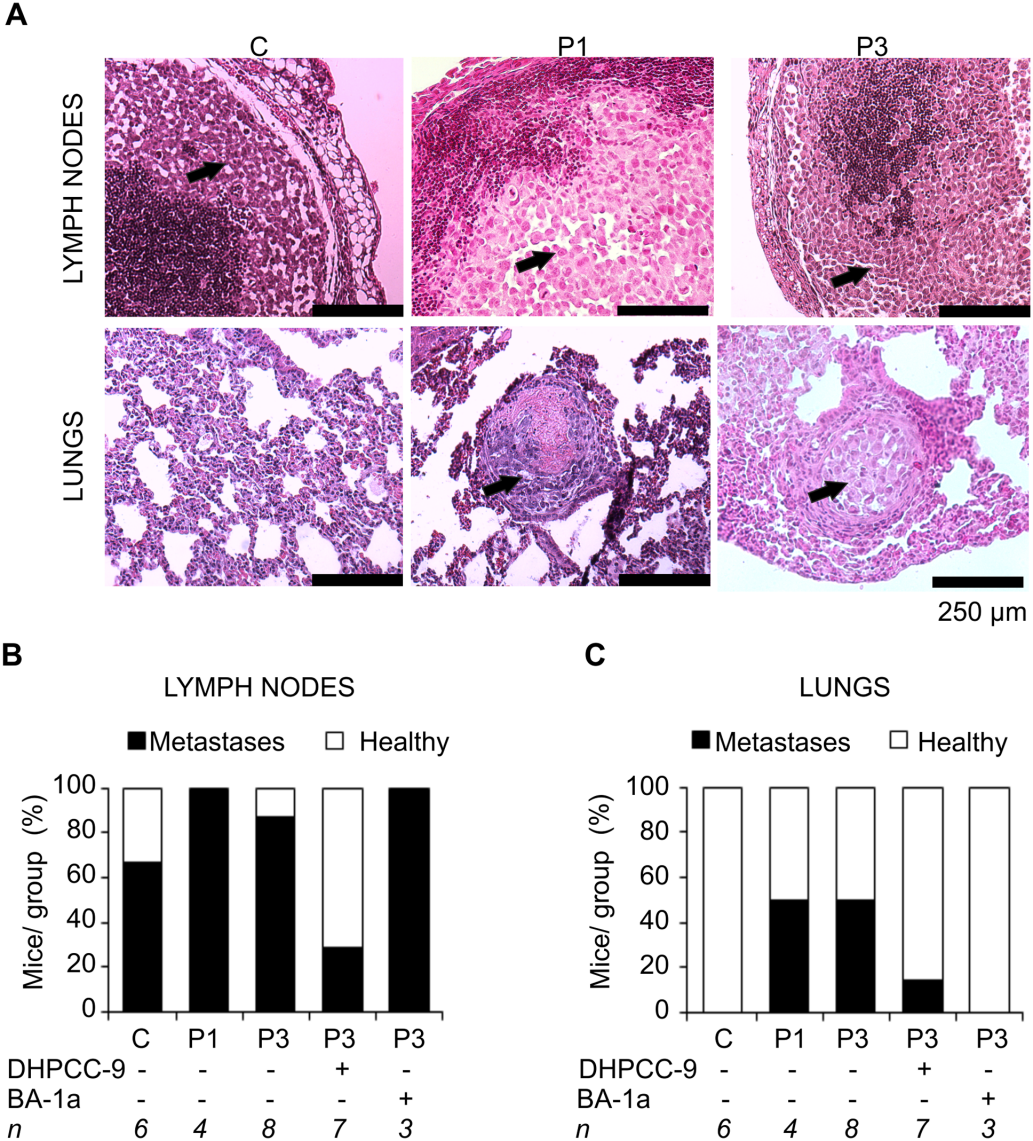
Pim inhibition by DHPCC-9 reduces the number of metastases in orthotopic prostate tumors overexpressing Pim-3. Different organs were collected from mice with orthotopic prostate tumor xenografts formed by PC-3 cells stably overexpressing an empty vector (C), Pim-1 (P1) or Pim-3 (P3). Paraffin-embedded tissue sections were stained with hematoxylin and eosin and analysed for the presence of metastases. Shown are representative images (A) from lymph node and lung sections (tumor cells indicated by arrows). The metastatic properties of xenografts from mice treated with DHPCC-9, BA-1a or their solvents were also analysed. Shown are percentages of mice positive for either lymph node metastases (B) or lung metastases (C) in each group.

Since the Tomato-derived fluorescence was not strong enough to clearly reveal the micrometastases (S6 Fig), histological analyses were carried out with tissue sections from kidneys, spleen, liver, lungs as well as the prostate-draining lymph nodes. After staining with haematoxylin and eosin, metastases were sought for from different tissue samples, but especially from lymph nodes and lungs. Intriguingly, while more than half of mock-transfected and most Pim-1 or Pim-3 xenografts had been able to metastasize into the prostate-draining lymph nodes, only Pim-overexpressing cells had invaded as far as into the lungs (Fig 3A–3C, S3 Table). Even more interestingly, DHPCC-9 treatment had efficiently inhibited formation of metastases in both organs. Both metastatic and necrotic areas were measured, but there was no clear connection to Pim activity (S6 Fig), suggesting that once a metastatic tumor is formed, the tumor cells may acquire other properties in addition to Pim activity to support their growth.

**Fig 4:**
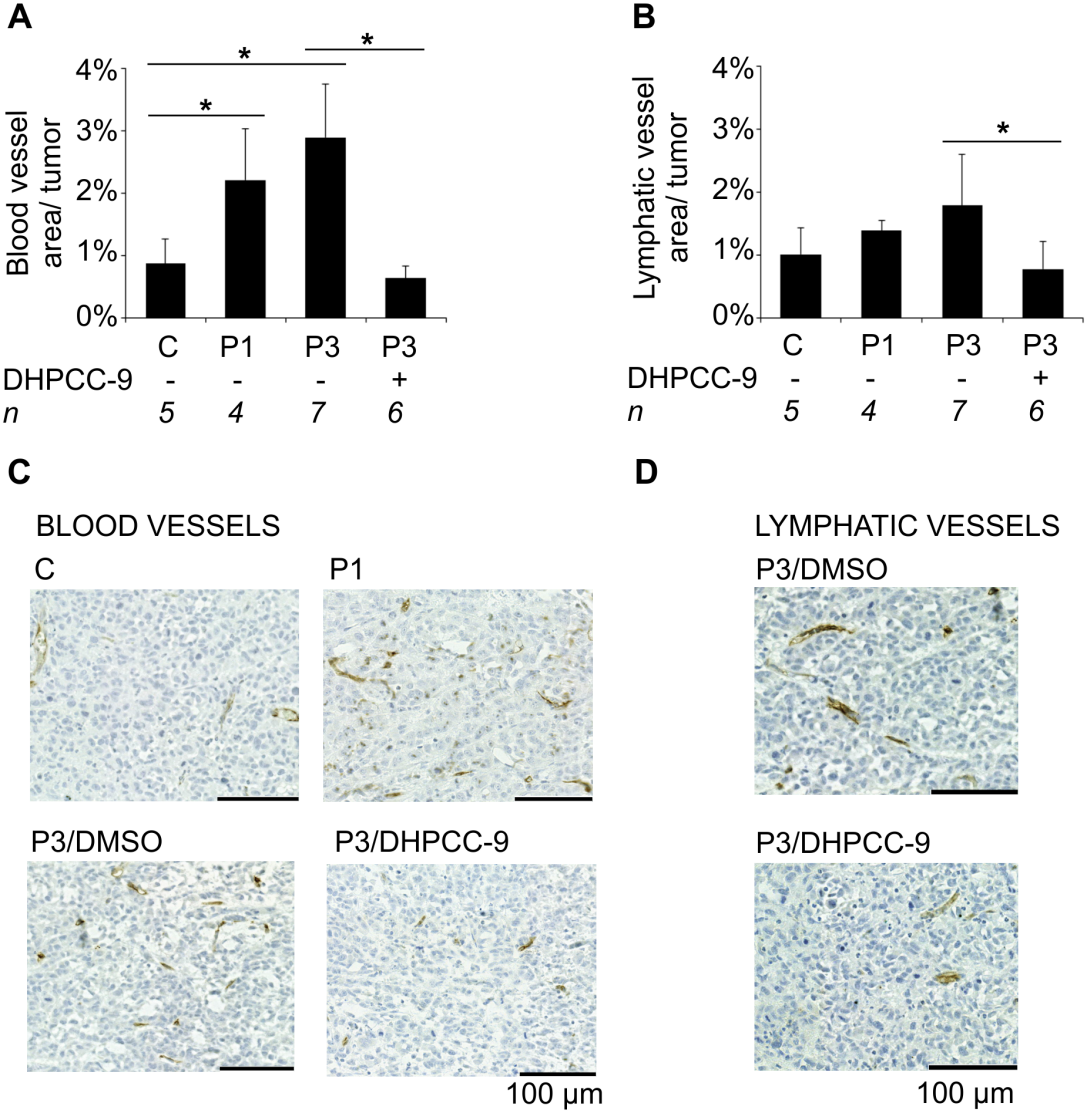
Pim kinases promote angiogenesis and lymphangiogenesis of prostate tumor xenografts. Angiogenic properties of the orthotopic prostate xenografts (Mock = C, Pim-1 = P1, Pim-3 = P3) were analysed by immunohistochemical staining of the paraffin-embedded tissue sections with anti-CD34 (blood vessels) and anti-m-LYVE-1 (lymphatic vessels) antibodies. Shown are average areas of all analysed blood (A) and lymphatic vessels (B) along with representative images (vessels in brown) (C-D) from fully imaged tumor tissue sections.

To visualize the vasculature of the orthotopic tumors, blood vessels and lymphatic vessels were stained. After quantitative analyses, a significant increase was detected in the areas of blood vessels per tumor in the xenografts formed by the Pim-overexpressing cells as compared to the control cells (Fig 4A). Slightly smaller differences were detected also in the areas of lymphatic vessels (Fig 4B). However, after treatment with the Pim inhibitor DHPCC-9, the areas of both blood as well as lymphatic vessels were significantly decreased (Fig 4A–4D).

### Pim-1 and Pim-3 enhance phosphorylation and cell surface expression of CXCR4

**Fig 5:**
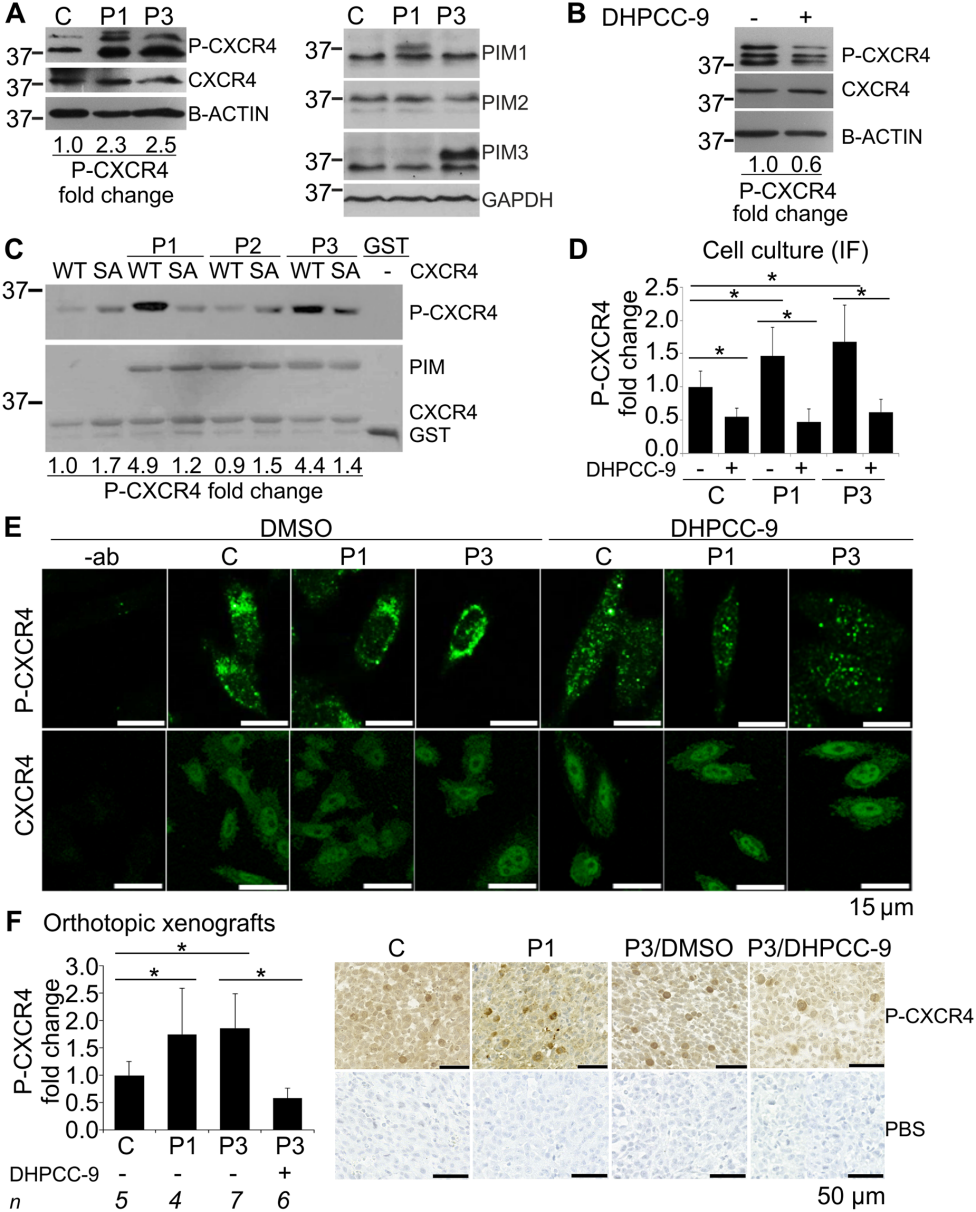
Pim-1 and Pim-3 enhance CXCR4 phosphorylation and cell surface expression in prostate cancer cells. Phosphorylation of CXCR4 at S339 as well as Pim levels were analysed by western blotting in the stable Pim-1 (P1), Pim-3 (P3) or control vector (C) overexpressing PC-3 cells or the parental PC-3 cell line treated with 0.1% DMSO or 10 μM DHPCC-9. Shown are results from one representative experiment with loading controls and molecular weight (kDa) markers (A-B). The ability of Pim family members to phosphorylate CXCR4 *in vitro* was analysed by incubating GST-tagged Pim-1 (P1), Pim-2 (P2) or Pim-3 (P3) proteins with GST-tagged fragments of wild-type (WT) or Ser339>Ala (SA) mutant human CXCR4. Phosphorylated CXCR4 was detected by phospho(Ser339)-CXCR4 antibody and protein loading by Ponceau S staining. Shown are results from one representative experiment (C). Localization and signal intensity of phosphorylated versus overall CXCR4 expression was analysed by immunofluorescent (IF) staining of stably transfected cells treated with either 0.1% DMSO or 10 μM DHPCC-9. The experiment was controlled by parallel samples stained only with the secondary antibody (-ab). Stainings were repeated twice and stacks of images were taken by confocal microscopy from at least 30 cells per sample per experiment. Shown are the signal intensities of phospho-CXCR4 stainings compared to overall CXCR4 levels along with representative images from phospho-CXCR4 and CXCR4 stainings (D-E). Phosphorylation and localization of CXCR4 was also analysed by immunohistochemical staining of the paraffin-embedded tissue sections from orthopic prostate tumors. Shown is the relative increase in the amount of phospho-CXCR4-positive cells versus overall CXCR4 expression measured by whole tumor scanning. PBS instead of the primary antibody was used as a negative control. Representative images were taken to visualize the differences in phospho-CXCR4 (dark brown) stainings (F).

The CXCR4 chemokine receptor protein has previously been implicated in PC-3 cell migration and interaction with endothelial cells [20–23]. Moreover, in hematopoietic cells Pim-1 has been shown to phosphorylate CXCR4 at Ser339, and thereby promote cell surface expression of CXCR4 and its interaction with the CXCL12 chemokine ligand [4]. In addition, we have previously shown Pim inhibition or silencing to efficiently reduce invasion of PC-3 cells towards MG-63 osteosarcoma cell conditioned medium, where the major chemoattractant is CXCL12 [6, 23]. To find out whether Pim/CXCR4 interaction plays a role also in our prostate cancer xenograft model, we first assessed whether there were differences in the phosphorylation status of CXCR4 between our stable cell lines. By Western blotting, we observed a marked increase in the Ser339-phosphorylated CXCR4 levels in both Pim-overexpressing stable cell lines as compared to the control cell line (Fig 5A). In addition, a decrease in phosphorylated CXCR4 levels was seen after treatment of parental PC-3 cells with the Pim inhibitor DHPCC-9 (Fig 5B).

Since stable overexpression of either Pim-1 or Pim-3 clearly enhanced CXCR4 phosphorylation, we wanted to compare the *in vitro* activities of all three Pim family members towards CXCR4. Therefore, we incubated GST-tagged Pim proteins together with GST-tagged C-terminal 46 aa fragments of CXCR4 (WT) or its mutated S339A form (SA), where the serine residue had been replaced by an alanine residue. When the *in vitro* phosphorylated fragments were detected with the anti-phospho(Ser339)-CXCR4 antibody, it became evident that both Pim-1 and Pim-3, but not Pim-2 can phosphorylate CXCR4 on Ser339 (Fig 5C). Similar differences were also detected in PC-3 cells after transient Pim overexpression, whereas the Pim inhibitor DHPCC-9 efficiently inhibited CXCR4 phosphorylation there (S8 Fig).

Thereafter we carried out immunofluorescence stainings to visualize the localization of CXCR4 protein in the stable PC-3 cell lines that had been treated for 24 h with DMSO or DHPCC-9. While CXCR4 was ubiquitously expressed, its phosphorylated form recognized by the anti-phospho(Ser339)-CXCR4 antibody displayed more membrane-associated expression in DMSO-treated samples, but rather dispersed and weaker cytoplasmic expression in DHPCC-9-treated samples (Fig 5D and 5E). While both Pim-1 and Pim-3 enhanced the phospho-CXCR4 signals as compared to overall CXCR4 levels, Pim inhibition efficiently reduced them, resulting also in slightly stronger nuclear expression of CXCR4 (Fig 5D and 5E).

To analyse the role of CXCR4 phosphorylation in our *in vivo* experiments, we used immunohistochemistry to measure the relative amounts of orthotopic prostate tumor cells positive for phosphorylated CXCR4. Comparison of phospho-CXCR4 signals to the average CXCR4 signals in each tumor revealed that both Pim-1 and Pim-3 had significantly increased the relative amounts of phospho-CXCR4, while Pim inhibition by DHPCC-9 had decreased it even below the level observed in the mock-transfected samples (Fig 5F). Altogether, both the *in vitro* and *in vivo* data suggest that tumors overexpressing Pim-1 or Pim-3 may take advantage of the CXCL12/CXCR4 chemokine pathway to spread into other organs such as the lungs.

## Discussion

Here we show that PC-3 prostate cancer cells overexpressing either Pim-1 or Pim-3 kinases form larger xenograft tumors than the parental PC-3 cells. These results are well in line with previous observations on the ability of Pim-1 and Pim-2 to enhance growth of PC-3 cell-derived subcutaneous prostate cancer xenografts [18], while here we demonstrate that Pim-3 is also equally effective. More intriguingly, when orthotopically inoculated into mouse prostates, cells overexpressing Pim-1 or Pim-3 have an increased capacity to metastasize from the prostate-based tumors to other organs such as the lungs. In addition, one of the tested Pim-inhibitory compounds, DHPCC-9, is able to decrease Pim-dependent tumor growth as well as formation of metastases without severe side effects, suggesting that it is able to penetrate into tumor cells to inactivate Pim kinases there.

While we have previously shown that Pim kinases are able to promote motility of prostate cancer cells [6], the present study is the first to demonstrate similar effects also under *in vivo* conditions. This is of interest, since orthotopically inoculated PC-3 cells have previously been shown to migrate from the prostate to the local prostate-draining sacral and iliac lymph nodes, but rarely anywhere else [24–26]. These findings were confirmed in our studies, where metastatic growth was observed in the prostate-draining lymph nodes of most tumor-bearing mice. However, only the Pim-overexpressing xenografts were able to metastasize into the lungs, while no metastases were detected in other collected tissues.

To address the tumorigenic mechanisms driven by Pim kinases and opposed by Pim inhibition, tissue samples from the primary xenograft tumors were analysed by immunohistochemistry for markers of mitotic activity, angiogenicity and invasiveness. Slight Pim-dependent increases were observed in the proportion of mitotic cells and in the areas of lymphatic vessels, while more significant upregulation was detected in the formation of tumor vasculature and in the phosphorylation and cell surface expression of CXCR4. By contrast, all these parameters were strongly decreased by the Pim-selective inhibitor DHPCC-9. Thus, these observations may explain not only the enhanced growth of tumors overexpressing Pim kinases, but also their metastatic properties.

The CXCL12/CXCR4 chemokine pathway is essential for lymphocyte trafficking and especially for homing of hematopoietic stem cells into the bone marrow [19, 27]. In addition, the CXCL12 chemokine is constitutively expressed in several other organs including lymph nodes and lungs and can thereby attract not only hematopoietic cells, but also migrating cancer cells that often show high expression levels of the CXCR4 receptor on their cell surface. Furthermore, CXCR4 can support tumor survival e.g. by promoting tumor vascularization.

Pim-1 overexpression has been associated with upregulated cell surface expression of CXCR4 in hematopoietic malignancies such as acute myeloid leukemia [4], diffuse large B-cell lymphoma [28] and chronic lymphocytic leukemia [29]. The intracellular tail of CXCR4 can be phosphorylated *in vitro* at Ser339 by Pim-1 kinase [4] and by Pim-3, as shown here, but not by Pim-2. Interestingly, Ser339 is among several serine residues targeted by G-protein receptor kinases (GRKs), resulting in receptor endocytosis [30]. By contrast, Pim-dependent phosphorylation of CXCR4 has been reported to lead to increased externalization of the receptor, allowing cells to migrate towards a CXCL12 gradient [4]. In PC-3 cells, CXCR4 surface levels are relatively low, unless the cells are treated with the CXCL12 ligand, which also clearly increases the invasive properties of these cells [20–23]. Thus, while additional studies on the effects of Pim kinases on CXCR4 in PC-3 cells cultured in the absence or presence of CXCL12 would be of interest, one can already speculate that the Pim-CXCR4 interaction has helped our Pim-overexpressing orthotopic tumor cells to form metastases into the prostate-draining lymph nodes and the lungs, while the tumor microenvironment around the subcutaneous tumors may not have been permissive enough to promote the invasion.

Since the CXCL12/CXCR4 pathway is an attractive therapeutic target, several small molecule compounds have been developed that either block the interaction of the chemokine with its receptor or inhibit signaling downstream from the receptor [19, 27]. Promising results have already been obtained from clinical trials that have aimed to increase chemosensitivity of hematopoietic malignancies, but CXCL12/CXCR4 inhibitors may also help to reduce the metastatic potential of solid cancers. One problem with these inhibitors is that they induce a counter-regulatory upregulation of CXCR4 on the cell surface, resulting in only short-lived responses. Therefore it might be more efficient to block externalization of the CXCR4 receptor by Pim-selective inhibitors that may also have less harmful side effects.

Here we have shown preliminary *in vivo* safety and efficacy data for one Pim-selective inhibitor, the pyrrolocarbazole DHPCC-9, whereas the benzo[*cd*]azulene BA-1a turned out to be too insoluble. DHPCC-9 did not show any cytotoxic effects in mice, even though some malformations were detected in the early-stage zebrafish embryos. These results suggest that at least a short-term inhibition of Pim activity can be well tolerated in adult organisms and that it may even be possible to use higher doses of this inhibitor to magnify the observed effects. It may also be advantageous to combine Pim inhibition with other treatments affecting cell survival or motility.

Data from cell-based wound healing experiments indicate that DHPCC-9 is able to block motility of PC-3 prostate cancer cells [6]. As shown also in this study, this is not simply due to decreased proliferation, since cell viability was not substantially reduced during the 24 h wound recovery follow-up period. However, longer exposure of PC-3 cells to DHPCC-9 reduced viable cell numbers in culture, which was well in line with the decreased tumor growth during the three-week test period. Yet for the prostate cancer patients, it is not the primary tumors but the metastases that are usually fatal. Therefore it will be important to be able to reduce the metastatic properties of the tumors e.g. by DHPCC-9-like compounds.

Even though the results with DHPCC-9 look promising, there are several obstacles to overcome if it was to be developed towards an actual drug compound. DHPCC-9 is soluble in DMSO, but this solvent is too toxic to be used within human patients. Therefore oral derivatives should be searched for. The putative cardiotoxicity of the compound should also be tested, since Pim kinases regulate cytokine responses and have essential functions in endothelial cells [31]. Here it should be noted that the first clinical Phase 1 trial with a Pim inhibitor, SGI-1776, had to be ended due to hERG channel toxicity [32]. However, its derivatives have displayed more favorable profiles, suggesting that the cardiotoxicity problems were due to the properties of the original compound, and not the Pim kinases targeted by it [32].

## Conclusions

Taken together, we have shown that Pim kinases play an important role in cancer progression by increasing the potential of tumor cells to grow as well as to invade not only to the surrounding tissues but also much further into the body. In addition to enhancing angiogenesis and lymphangiogenesis, Pim kinases are likely to promote metastatic prostate cancer growth by employing the CXCL12/CXCR4 chemokine pathway. Furthermore, we have provided preliminary evidence for the safety and efficacy of the Pim-selective inhibitor DHPCC-9 as a promising compound to decrease Pim-induced cell proliferation and motility also *in vivo*. Such compounds are clearly needed to combat the fatal metastases associated with prostate cancer and other solid tumors.

## Materials and Methods

### Cell culture and transfections

The human androgen-independent prostate epithelial adenocarcinoma cell line PC-3 (American Type Culture Collection) was maintained in Roswell Park Memorial Institute (RPMI) -1640 medium, supplemented with 10% fetal bovine serum, L-glutamine and antibiotics. To prepare PC-3-derived cell lines stably overexpressing human Pim family members, PC-3 cells were transfected with pcDNA3.1/V5-His-C-based expression vectors for Pim-1 or Pim-3 (kindly provided by Markku Varjosalo, University of Helsinki, Finland) or mock-transfected with the empty vector (Thermo Fisher Scientific, Waltham, MA, USA). In addition, all cells were cotransfected with the pCMV-Td-Tomato plasmid (Clontech Laboratories Inc., Mountain View, CA, USA) to be used as a fluorescent follow-up marker. All transfections were performed with Fugene 6 (Promega, Madison, WI) in 3:1 ratio to DNA. After an overnight incubation, positively transfected cells were enriched by antibiotic selection with G418 (Fisher Scientific, Geel, Belgium), first using 300 μg/ml for 48 h and thereafter 500 μg/ml for 14 days. Medium was changed every day during the selection. After selection, maintenance of the transfected plasmids in the stable cell lines generated from pools of cells was ensured by supplementing culture medium with 200 μg/ml of G418.

### Kinase inhibitors

Two structurally distinct types of Pim-selective inhibitors were used that have been described and validated before: the pyrrolozarbazole DHPCC-9 (1,10-dihydropyrrolo[2,3-*a*]carbazole-3-carbaldehyde) [6, 15] and the benzo[*cd*]azulenes BA-1a and BA-2c [16, 17].

### Migration assays

Cells were plated on 24-well plates (100 000 cells/well). After 24 or 48 hours, confluent cell layers were scratched with 10 μl pipette tips. Thereafter, the cells were treated with 10 μM DHPCC-9 or BA-1a or control-treated with DMSO (0.1%). Images were taken by 20× magnification at indicated time-points. The width of the wound was analysed by ImageJ software (1.37v, Wayne Rasband, National Institute of Health, Bethesda, MD, USA) by manually drawing the wound lines and analysing the wound area in square pixels.

### Viability assays

Cells were plated on 96-well plates (35 000 cells/ well). After attachment, the cells were treated with 10 μM DHPCC-9 or BA-1a or control-treated with DMSO (0.1%). Cell viability was analysed by MTT assay as previously described [6].

### Toxicity assays with zebrafish embryos

Housing and experiments of wild-type zebrafish (*Danio rerio*) were performed according to the European Convention for the Protection of Vertebrate Animals used for Experimental and other Scientific Purposes, and the Statutes 1076/85 and 62/2006 of The Animal Protection Law in Finland and EU Directive 86/609. Briefly, the animals were maintained at 26°C according to standard procedures [33] in the aquatic facilities of the Laboratory of Animal Physiology, University of Turku, Finland under the licence ID ESAVI/4068/04.10.07/2013 from the Provincial State Office of Western Finland. Breeding traps were placed in the fish tanks and after natural spawning, the fertilized embryos were collected and cultured in E3 medium (5 mM NaCl, 0.17 mM KCl, 0.33 mM CaCl_2_, 0.33 mM MgSO_4_, 0.01% methylene blue) at 28°C. Treatments with Pim inhibitors or 0.1% DMSO (control) were started at 6 h post-fertilization (hpf). Toxicity was assessed by scoring embryos as live or dead at 26 hpf under stereomicroscope (Zeiss StereoLumar V.12, Carl Zeiss Microscopy GmBH, Jena Germany). For more detailed morphological analysis, embryos treated with DMSO or DHPCC-9 embryos were dechorionated and imaged with stereomicroscope at 50 hpf. Embryo length was measured as greatest length from head to tail. The developmental stage (head-trunk angle) was measured as described earlier [34]. Body curvature was measured as an angle between center line of notochord extending to the level of the posterior end of yolk sac extension (yolk-anus-tail angle), and as a line from this point to the most posterior end of the last somite. Pericardiac oedema was quantitated by measuring the area of pericardiac space. All image analyses were performed using ImageJ software (1.48s, Fiji, Wayne Rasband, National Institutes of Health, Bethesda, MD, USA).

### Toxicity assays with mice

All mouse experiments were carried out at the Central Animal Laboratory of the University of Turku, Finland according to the European Convention for the Protection of Vertebrate Animals used for Experimental and other Scientific Purposes, and the Statutes 1076/85 and 1360/90 of The Animal Protection Law in Finland and EU Directive 86/609. Accordingly, the clinical signs of mice were daily recorded, and if the criteria of humane endpoints were met, animals were sacrificed. Humane endpoints were considered as rapid or gradual weight loss, abnormal changes in behavior and motion (social and eating behavior), subcutanic tumor size greater than 1.5 cm in diameter or skin problems (wounds or signs of inflammation). The experimental procedures were reviewed by the local Ethics Committee on Animal Experimentation of the University of Turku and approved by the Provincial State Office of Western Finland with the licence IDs ESAVI/2008-05531 and ESAVI/3937/04.10.03/2011. Two available batches of mice from Harlan Laboratories (Horst, the Netherlands) were initially used, males (FVB/NhanHsd) and females (BALB/cOlaHsd), and maintained under controlled conditions (20–21°C, 30–60% relative humidity and 12-hour lighting cycle).

DHPCC-9 was dissolved in DMSO and intraperitoneally injected in 20 μl total volume of DMSO into FVB/NHanHsd male mice in concentration of 100 mg/kg/day for two days and thereafter 50 mg/kg/day for 8 days. BA-1a was dissolved in DMA and intraperitoneally injected into BALB/cOlaHsd female mice, either 25 mg/kg/day in 25 μl of total volume of DMA for 6 days or 10–20 mg/kg/day in 10 μl of total volume of DMA for 17 days. Animal welfare and weight was monitored daily until the mice were anesthesized by CO_2_ and sacrificed. Tissue samples from liver, spleen and kidneys were collected to search for possible abnormalities.

### Efficacy assays with xenografted mice

The follow-up periods of both subcutaneous and orthotopic experiments were designed according to previous PC-3 xenograft studies [18, 25–26]. For subcutaneous inoculations, PC-3 cells stably transfected with Pim-1, Pim-3 or the empty vector were collected, while cells were growing in a logarithmic phase. The cells were suspended in sterile PBS (4.5× 10^6^ cells in 100 μl) and injected into both flanks of athymic nude male mice (Balb/cOlaHsd-Foxn1nu/nu, Harlan Laboratories, Horst, the Netherlands), which were maintained in controlled and pathogen-free environment. Animal welfare was monitored daily, and animals were weighed and tumors palpated every other day. Tumor volume was calculated according to the formula V = (π/6)(d_1_ × d_2_)^3/2^ (20), where d_1_ and d_2_ are perpendicular tumor diameters (width, length). The fluorescently labelled tumor cells were imaged by IVIS Lumina II (Xenogen corp./ Caliper Life Sciences, Inc., Hopkinton, MA, USA) at different time points during the experiment, after which tumor areas (square pixels) and average signal intensities were measured by ImageJ. After three weeks, mice were sacrificed and tumors as well as selected tissues (kidneys, spleen, liver, lungs and prostate-draining lymph nodes) were collected.

For orthotopic inoculations, cells were suspended in sterile PBS (Biochrom AG, Berlin, Germany; 10^6^ cells in 20 μl) containing green food color 33022 (5 μg/ml; Roberts Oy, Turku, Finland) and kept on ice until usage. Cells were inoculated into the ventral prostates of anesthetized mice as previously described [25]. Analgesic drug Temgesic (Reckitt Benckiser Healthcare Ltd, Hull, UK) was given to mice prior to operation, 24 h and 48 h after them and also during the three-week follow-up period when needed.

Two separate orthotopical sets of experiments were performed. In the second set, part of the mice inoculated with Pim-3-overexpressing cells were daily treated with intraperitoneal injections of either 50 mg/kg of DHPCC-9 in 20 μl of DMSO or 20 mg/kg of BA-1a in 10 μl of DMA or equal amounts of the dissolvents as controls. All treatments were initiated one day after the orthotopic inoculations. Animal welfare and weight was monitored daily until the mice were sacrificed, after which the tumors as well as tissue samples were first imaged by IVIS Lumina II (Xenogen corp./ Caliper Life Sciences, Inc., Hopkinton, MA, USA) and then collected and stored for further analysis as described below. Fluorescent signals in each animal or isolated organ were normalized according to background signals given by tissues not expected to contain metastases or by signals originating from food. Tumor volume was calculated according to the formula V = (π/6)(d_1_ × d_2_ × d_3_), where d_1—_d_3_ are perpendicular tumor diameters (width, length, height) [35].

### Histology and immunohistochemistry

Tumors and tissue samples were fixed for 24 h in 4% paraformaldehyde, after which they were stored in 70% EtOH. After paraffin embedding, 5 μm sections were cut and sections were stored at +4°C until they were deparaffinized, stained and rehydrated. All tumor and tissue samples were first stained by Mayer’s Hematoxylin and Eosin (H&E). Additional immunohistochemical stainings were performed to visualize mitotic cells, expression of V5-tagged constructs, blood vessels, lymphatic vessels and phosphorylation of CXCR4 (S4 Table). For each staining, paraffin-embedded tissue sections were deparaffinized, microwaved, washed in water and blocked in 3% hydrogen peroxide in methanol. Samples were washed first in water and then in PBS or TBS, after which they were blocked and stained with antibodies. In addition, sections were counterstained by dipping them for 5–10 s in Mayer’s hematoxylin, after which samples were washed in water and dehydrated. As a negative staining control, primary antibody was replaced by PBS or TBS in each sample. Stable PC-3/pcDNA3.1-VEGF-C tumor tissue [26] was used as a negative control for V5 staining.

To analyse the stainings representative images were taken by Leica DMRXA microscope (Leica Microsystems CMS GmbH, Mannheim, Germany) and ISCapture V2.6. software (Xintu Photonics Co., Ltd, Tucsen, Fuzhou, China), while whole tumor scans were performed either by Olympus BX51 microscope with DotSlide software (Olympus Corporation, Tokyo, Japan) or the Pannoramic 250 slide scanner with Pannoramic Viewer (3DHistech Ltd., Budapest, Hungary). Images were further analysed by ImageJ. For analysis of signal intensities and stained areas, a color deconvolution by H&E DAB was performed, then images were turned into grayscale and colors were inverted, background was subtracted and threshold levels were adjusted. Thereafter particles were analysed. For other than vessel analyses, necrotic areas were avoided. In addition, fully necrotic tumors were left out from analyses (one fully necrotic tumor/ each group except for none among PC-3/Pim-1-derived tumors, S5 Table).

### Western blotting

Cells were washed once with PBS and resuspended in 2x Laemmli Sample Buffer. Samples were vortexed for 5 s and heated at 95°C for 5 min. Protein samples were then separated by SDS-PAGE, immobilized onto PVDF-membrane (Merck Millipore, Billerica, MA, USA) and incubated with primary antibodies (S6 Table). Signal was created using mouse (#7076, Cell Signaling Technology, 1:5000) or rabbit (#7074, Cell Signaling Technology, 1:5000) HRP-linked secondary antibodies and Amersham ECL Plus or Prime (GE Healthcare, Fairfield, CT, USA) or Pierce ECL (Thermo Fisher Scientific Inc.) chemiluminescence reagents. In addition, for analysing the phospho-CXCR4 levels, the signal intensities were calculated by ChemiDoc MP System with Image Lab software (Bio-Rad Laboratories, Inc., Hercules, CA, USA). Thereafter the phospho-CXCR4 signal values were compared to overall CXCR4 values.

### *In vitro* phosphorylation assays

GST-tagged constructs expressing human CXCR4 C46-WT and C46-S339A fragments [4] were kindly provided by Alex Bullock (University of Oxford, Oxford, UK). These fragments as well as full-length human Pim-1, human Pim-2 and mouse Pim-3 proteins were produced in bacteria, purified and analysed by *in vitro* phosphorylation assays as previously described [17] except that no radioactively labelled ATP was used. Samples were separated on SDS-PAGE, after which Western blotting with anti-phospho(Ser339)-CXCR4 was used to detect CXCR4 phosphorylation. Protein loading was analysed from PVDF-membrane by staining with Ponceau S solution (Sigma, St.Louis, MO, USA). Signal intensities were analysed by the ChemiDoc MP System.

### Immunofluorescence

For confocal microscopy, cells were plated on coverslips on 12-well plates (100 000 cells/well). After 24 hours, cells were treated with DMSO or the Pim inhibitor DHPCC-9 (10 μM). After another 24 h incubation, samples were fixed in 4% PFA, permeabilized in 0,25% Triton X-100/PBS and blocked in 1% BSA for 30 min at +37°C. Thereafter samples were stained with anti-phospho(Ser339)-CXCR4 or anti-CXCR4 antibodies (1:1000) overnight. For secondary antibody, Alexa-Fluor 488-labelled chicken anti-rabbit IgG (H+L) antibody (A21441, Life Technologies, 1:1000) was used for 30 min at +37°C and 30 min at RT. Cells were imaged by Leica DMRXA TCS SP5 Matrix confocal microscope with LAS AF Application (Leica Microsystems CMS GmbH, Mannheim, Germany). Signal intensities were analysed by ImageJ.

### Statistical analyses

Statistical analyses were performed by one-way ANOVA variance analyses with LSD post hoc multiple comparison tests (IBM SPSS Statistics 22, Chicago, Illinois, USA). In addition, Microsoft Excel data analysis tool t-Test: Two-Sample Assuming Unequal Variances was used in the supplementary assays. Pearson’s correlations were determined by Microsoft Excel data analysis tools and interpreted according to common quidelines [36]. The mean differences of ≤0.05 were considered significant. The graphs with means and standard deviations have been produced by Microsoft Excel.

## Supporting Information

S1 Fig Subcutaneous tumor imaging and cell culture control. PC-3-derived cell lines that had been stably transfected with the fluorescent Tomato vector and an empty vector (C) or a vector expressing Pim-3 (P3) were subcutaneously inoculated into the left and right backsides of nude mice (n = 4 +4). During the test period of 24 days, manual palpation and fluorescent imaging were perfomed three times. Correlations were analysed between the manually measured tumor volumes and either the areas or average signal intensities measured by fluorescent scanning. Shown are average results per each mouse at every time point (A). For analysis of mitotic cells, phospho-histone H3 staining (brown) was performed. Shown are representative images from control (C) and Pim-3 (P3) overexpressing tumors as well as whole tumor scan images for visualization of the differences in tumor size (B-C). Simultaneously to the animal experiment, cells were cultured in the absence of antibiotic selection to confirm stability of Pim-3 overexpression during the three-week test period (D).
(TIF)

S2 Fig Both DHPCC-9 and BA-1a Pim inhibitors decrease migration and viability of stable Pim-overexpressing PC-3 cells. Cell motility of stable control (C), Pim-1 (P1) or Pim-3 (P3) overexpressing PC-3 prostate cancer cells was analysed by wound healing assays. Cells were cultured on 24-well plates until confluency, after which wounds were scratched with 10 μl pipette tips. Cells were treated with 0.1% DMSO or DMSO-dissolved Pim inhibitors and samples were imaged and analysed at 0 and 24 h time-points. Shown are representative images along with average values from cells treated with either DHPCC-9 (A) or BA-1a (B). After 24 and 72 hours, viability of the cells was analysed by MTT assays. Shown are average OD_570_ values from triplicate samples from one representative experiment (C). For each assay, at least three separate experiments were carried out with highly similar results.
(TIF)

S3 Fig DHPCC-9 tolerance in zebrafish embryos. Zebrafish embryos were treated at 6 h post-fertilization and analysed at 50 h post-fertilization. Shown is average survival in two experiments (A), and body curvatures (B-C) as well as pericardial sac sizes (D) in one experiment with representative images to visualize the angles and the pericardiac sac indicated by an arrow.
(TIF)

S4 Fig Mouse weight gain during toxicity testing. White male or female mice were treated with various concentrations of either DMSO (A) or DMA (B-C) diluted Pim inhibitors and followed up for indicated time-periods to gain information about the possible cytotoxicity of the compounds.
(TIF)

S5 Fig Mouse weight gain during the second orthotopic experiment. Stable control (C) or Pim-1 (P1) or Pim-3 (P3) overexpressing PC-3 cells were orthotopically inoculated into nude mice. Mice were treated with DMSO or DMA as a control or with Pim inhibitors DHPCC-9 or BA-1a. Shown is the average mouse weight gain in each group during the test period.
(TIF)

S6 Fig Fluorescent imaging of the second orthotopic set tissue samples. At the second orthotopic set, tumors and tissue samples were fluorescently imaged to obtain information on the Tomato-derived signal of stably transfected PC-3 cells (Mock = C, Pim-1 = P1, Pim-3 = P3). Mice with control or Pim-3-overexpressing tumors were treated with 50 mg/kg of DHPCC9 in DMSO or 20 mg/kg of BA-1a in DMA or vehicles only. After approximately three weeks, mice were sacrificed and tissues were imaged. In each animal, signal intensity was normalized according to background signal given by a kidney. Lymph nodes are pointed out by arrows. Shown are images from tumors and collected tissue samples (A). After detection of metastases in the lymph node and lung sections, the average areas of the metastases and the average necrotic areas in them were analysed. Shown are areas as well as the number (n) of mice with metastases in control treated and DHPCC-9 treated animals (B).
(TIF)

S7 Fig V5-immunostaining of xenografted cells within orthotopic tumors and their lymph node metastases. Paraffin-embedded tissue sections from the second orthotopic set of tumors (Mock = C, Pim-1 = P1 and Pim-3 = P3), their surrounding mouse tissues and one control tumor (Neg. Ctrl) were stained with anti-V5 antibody. Shown are representative images from V5-positive or—negative samples.
(TIF)

S8 Fig Pim-1 and Pim-3 increase and DHPCC-9 decreases CXCR4 phosphorylation in PC-3 cells. PC-3 cells transiently overexpressing an empty vector (C), Pim-1 (P1), Pim-2 (P2) or Pim-3 (P3) were treated with DMSO or 10 μM DHPCC-9 for 24 hours. CXCR4 phosphorylation was detected by phospho(Ser339)-CXCR4 antibody, after which the signal intensity was compared to the intensity of the CXCR4 signal. Pim overexpression was confirmed by Pim-specific antibodies, while β-actin was used as a loading control.
(TIF)

S1 Table Pim inhibitor tolerance in zebrafish embryos. List of zebrafish embryos treated with Pim inhibitors or DMSO at 6 h post-fertilization and analysed for their viability and possible abnormalities at 50 h post-fertilization.
(XLSX)

S2 Table Animal numbers in the orthotopic experiments. List of mice with or without prostate xenograft tumors derived from the stable PC-3 cell lines in the presence of control (DMSO or DMA) or Pim inhibitor treatments.
(XLSX)

S3 Table Metastases from orthotopic tumors. List of mice with prostate xenograft tumors and metastases in the prostate-draining lymph nodes and/or the lungs. In addition, the groups were compared based on the number of metastases in different organ types (lymph nodes or/and lungs). DMSO or DMA treatments have been combined as control treatments.
(XLSX)

S4 Table Protocols for immunohistochemistry. Immunohistochemical staining of paraffin-embedded tissue samples was done according to following protocols for antigen retrieval (1), blocking (2), primary (3) and secondary antibody stainings (4), Avidin-Biotin reaction (5) and DAB reaction (6).
(XLSX)

S5 Table Detailed information for excluding samples in immunohistochemical analysis of the second orthotopic set samples. Original sample number represents the number of mice with tumors in each group. DMSO or DMA treatments are combined as control treatments.
(XLSX)

S6 Table Antibody dilutions for Western blotting. Table contains details for primary antibody dilutions incubated at +4°C overnight.
(XLSX)
