# Supplementary figures and images for: Pim Kinases Promote Migration and Metastatic Growth of Prostate Cancer Xenografts
Source: PLoS One. 2015 Jun 15;10(6):e0130340. doi: 10.1371/journal.pone.0130340 (PMC4467846; doi:10.1371/journal.pone.0130340)

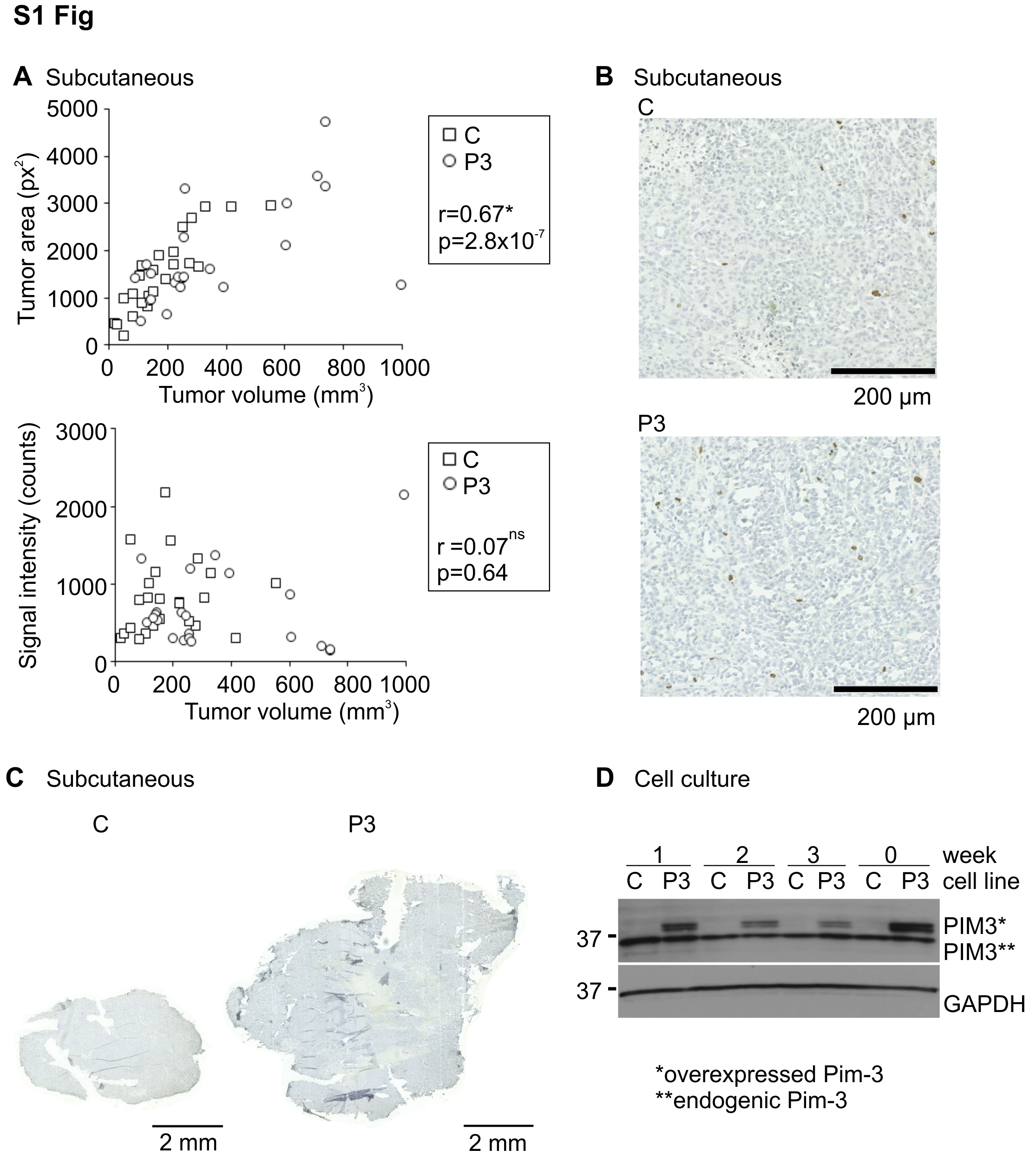

Supplement: S1 Fig — PC-3-derived cell lines that had been stably transfected with the fluorescent Tomato vector and an empty vector (C) or a vector expressing Pim-3 (P3) were subcutaneously inoculated into the left and right backsides of nude mice (n = 4 +4). During the test period of 24 days, manual palpation and fluorescent imaging were perfomed three times. Correlations were analysed between the manually measured tumor volumes and either the areas or average signal intensities measured by fluorescent scanning. Shown are average results per each mouse at every time point (A). For analysis of mitotic cells, phospho-histone H3 staining (brown) was performed. Shown are representative images from control (C) and Pim-3 (P3) overexpressing tumors as well as whole tumor scan images for visualization of the differences in tumor size (B-C). Simultaneously to the animal experiment, cells were cultured in the absence of antibiotic selection to confirm stability of Pim-3 overexpression during the three-week test period (D). (TIF) [file pone.0130340.s001.tif]

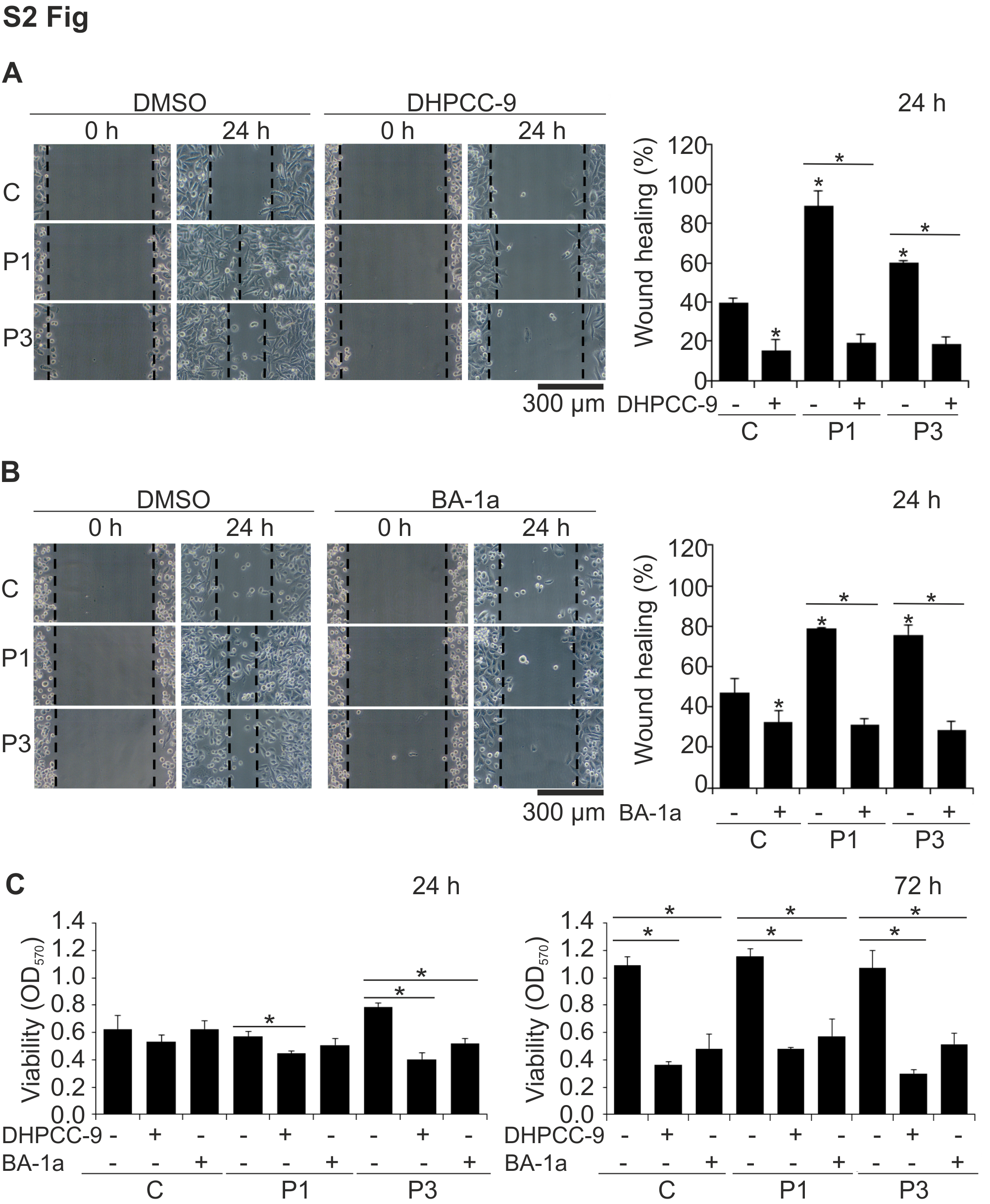

Supplement: S2 Fig — Cell motility of stable control (C), Pim-1 (P1) or Pim-3 (P3) overexpressing PC-3 prostate cancer cells was analysed by wound healing assays. Cells were cultured on 24-well plates until confluency, after which wounds were scratched with 10 μl pipette tips. Cells were treated with 0.1% DMSO or DMSO-dissolved Pim inhibitors and samples were imaged and analysed at 0 and 24 h time-points. Shown are representative images along with average values from cells treated with either DHPCC-9 (A) or BA-1a (B). After 24 and 72 hours, viability of the cells was analysed by MTT assays. Shown are average OD570 values from triplicate samples from one representative experiment (C). For each assay, at least three separate experiments were carried out with highly similar results. (TIF) [file pone.0130340.s002.tif]

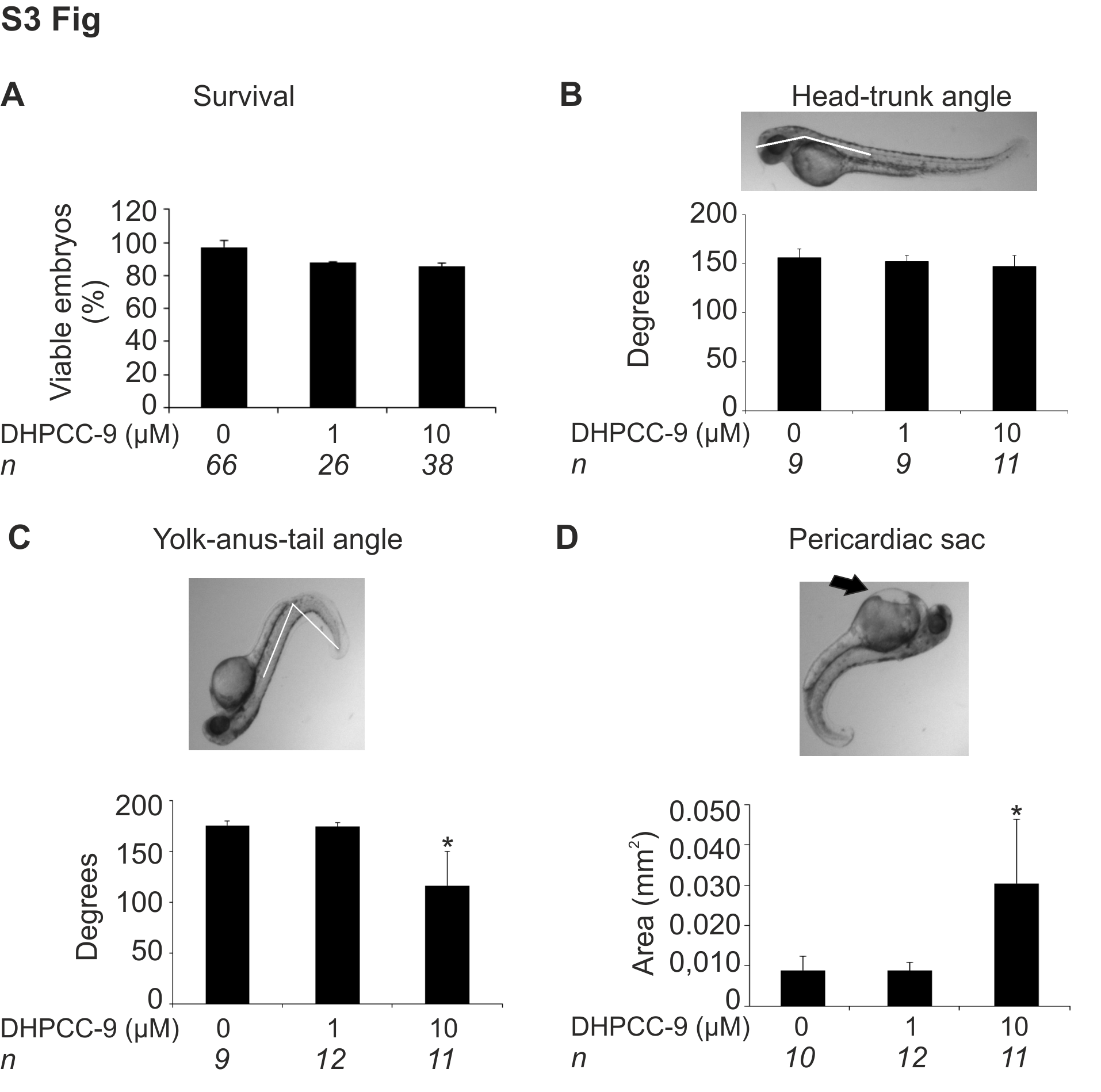

Supplement: S3 Fig — Zebrafish embryos were treated at 6 h post-fertilization and analysed at 50 h post-fertilization. Shown is average survival in two experiments (A), and body curvatures (B-C) as well as pericardial sac sizes (D) in one experiment with representative images to visualize the angles and the pericardiac sac indicated by an arrow. (TIF) [file pone.0130340.s003.tif]

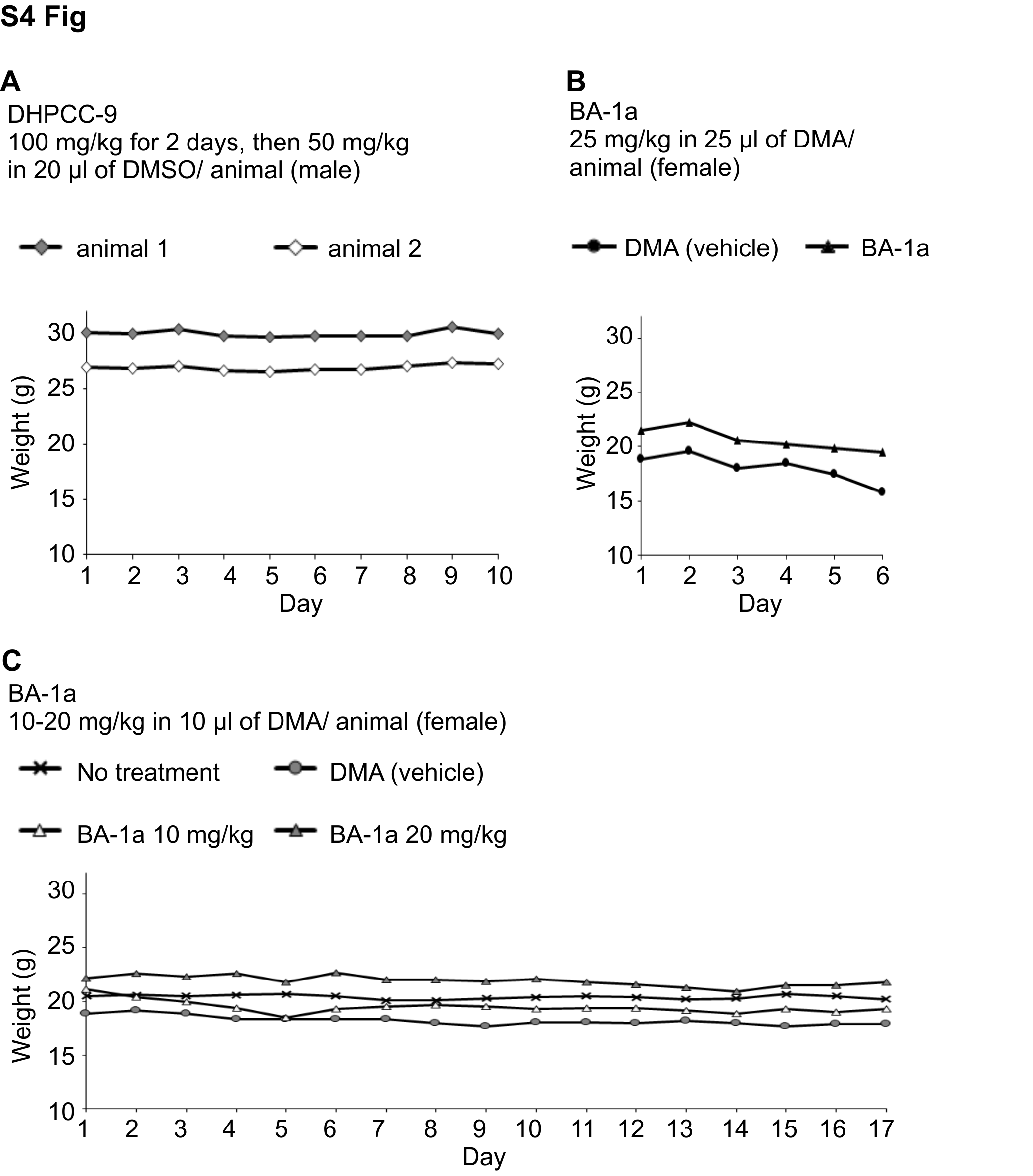

Supplement: S4 Fig — White male or female mice were treated with various concentrations of either DMSO (A) or DMA (B-C) diluted Pim inhibitors and followed up for indicated time-periods to gain information about the possible cytotoxicity of the compounds. (TIF) [file pone.0130340.s004.tif]

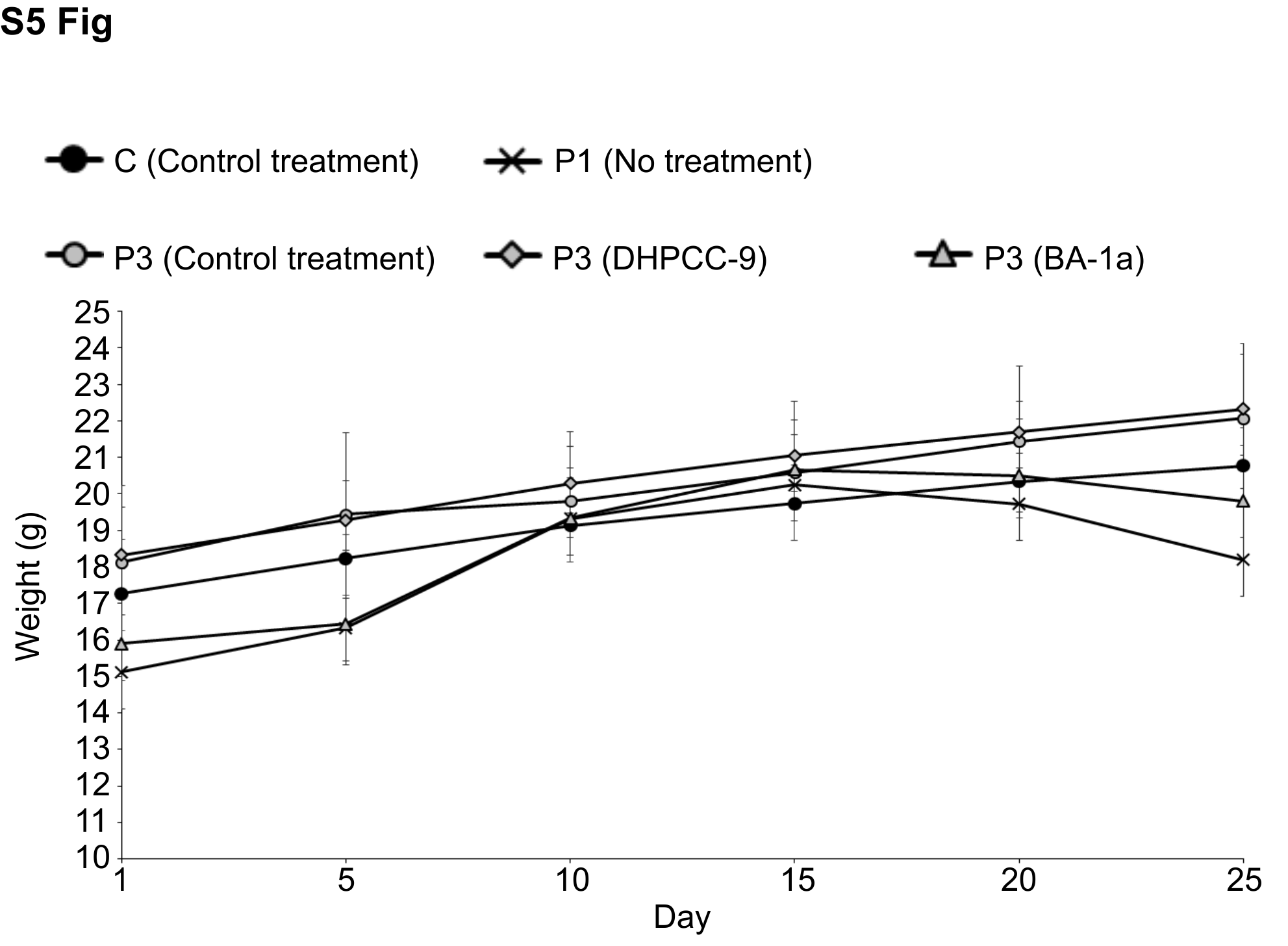

Supplement: S5 Fig — Stable control (C) or Pim-1 (P1) or Pim-3 (P3) overexpressing PC-3 cells were orthotopically inoculated into nude mice. Mice were treated with DMSO or DMA as a control or with Pim inhibitors DHPCC-9 or BA-1a. Shown is the average mouse weight gain in each group during the test period. (TIF) [file pone.0130340.s005.tif]

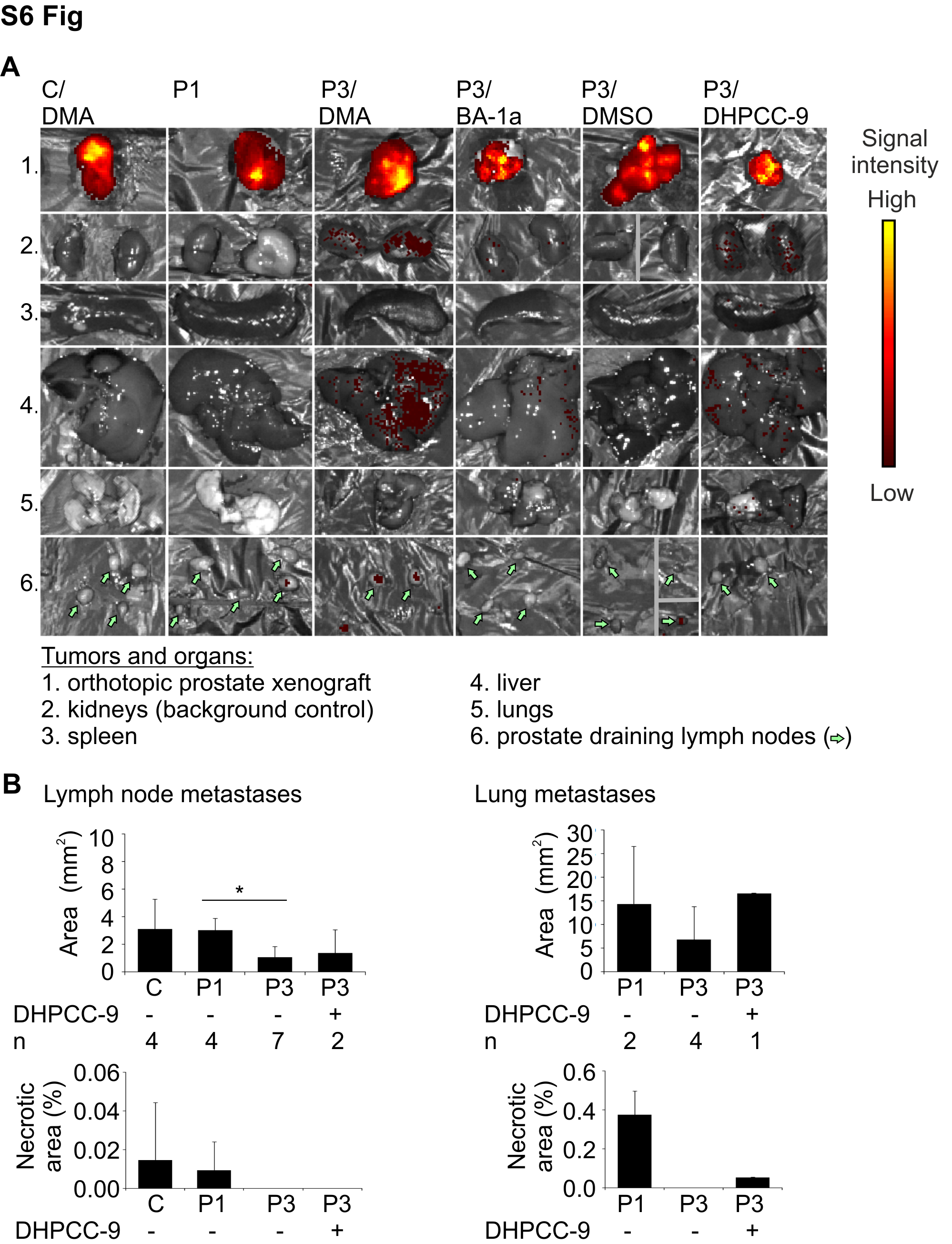

Supplement: S6 Fig — At the second orthotopic set, tumors and tissue samples were fluorescently imaged to obtain information on the Tomato-derived signal of stably transfected PC-3 cells (Mock = C, Pim-1 = P1, Pim-3 = P3). Mice with control or Pim-3-overexpressing tumors were treated with 50 mg/kg of DHPCC9 in DMSO or 20 mg/kg of BA-1a in DMA or vehicles only. After approximately three weeks, mice were sacrificed and tissues were imaged. In each animal, signal intensity was normalized according to background signal given by a kidney. Lymph nodes are pointed out by arrows. Shown are images from tumors and collected tissue samples (A). After detection of metastases in the lymph node and lung sections, the average areas of the metastases and the average necrotic areas in them were analysed. Shown are areas as well as the number (n) of mice with metastases in control treated and DHPCC-9 treated animals (B). (TIF) [file pone.0130340.s006.tif]

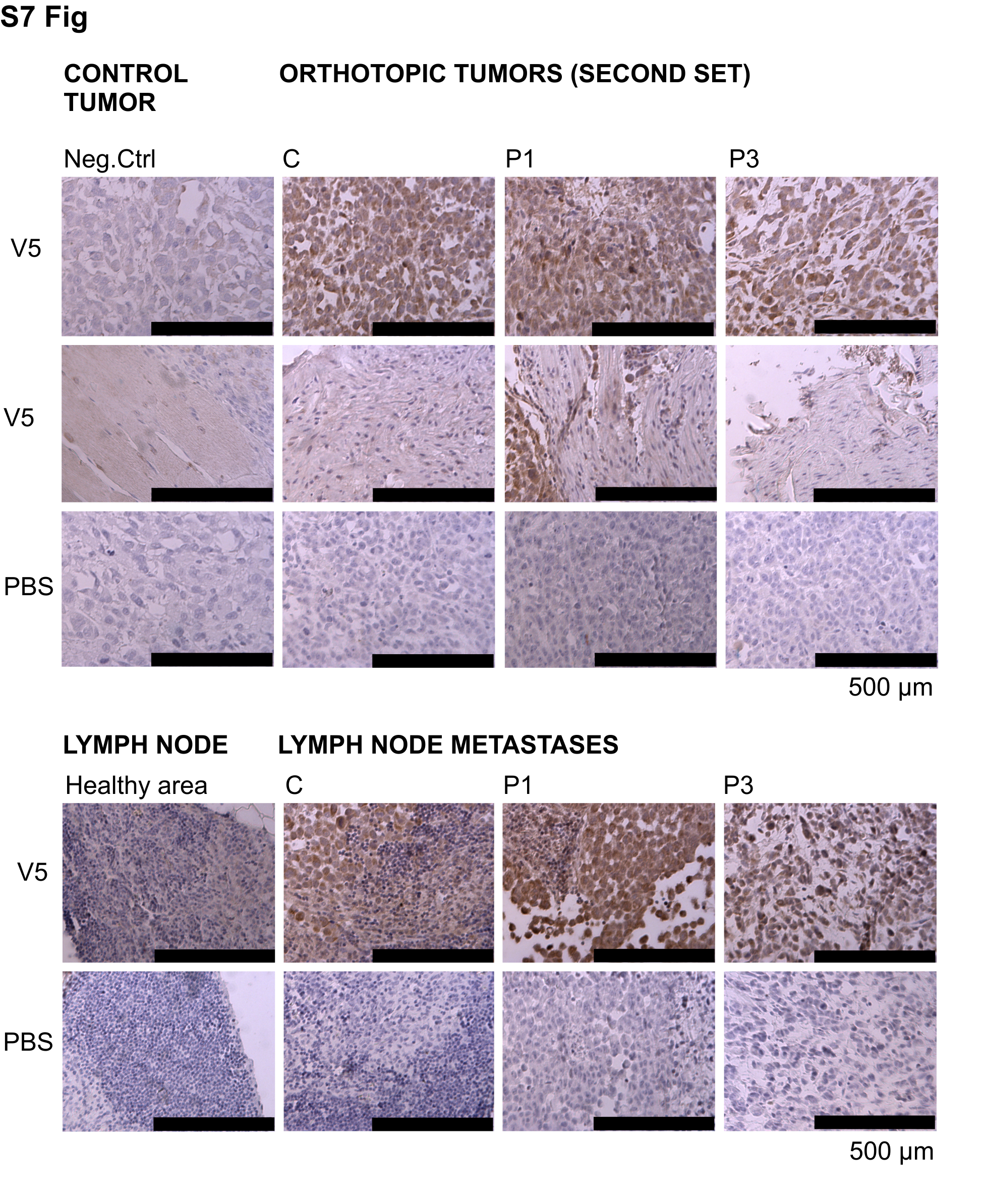

Supplement: S7 Fig — Paraffin-embedded tissue sections from the second orthotopic set of tumors (Mock = C, Pim-1 = P1 and Pim-3 = P3), their surrounding mouse tissues and one control tumor (Neg. Ctrl) were stained with anti-V5 antibody. Shown are representative images from V5-positive or—negative samples. (TIF) [file pone.0130340.s007.tif]

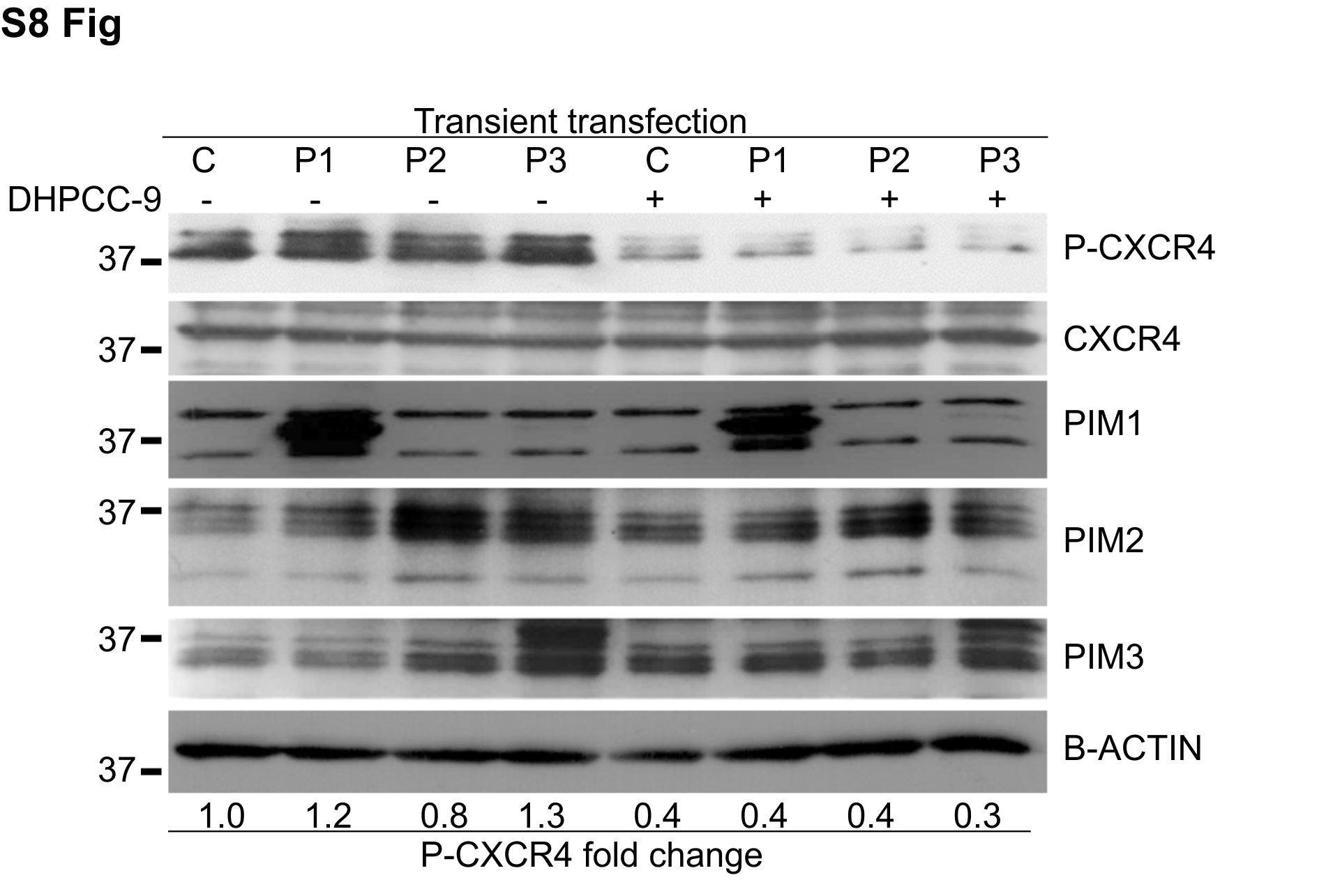

Supplement: S8 Fig — PC-3 cells transiently overexpressing an empty vector (C), Pim-1 (P1), Pim-2 (P2) or Pim-3 (P3) were treated with DMSO or 10 μM DHPCC-9 for 24 hours. CXCR4 phosphorylation was detected by phospho(Ser339)-CXCR4 antibody, after which the signal intensity was compared to the intensity of the CXCR4 signal. Pim overexpression was confirmed by Pim-specific antibodies, while β-actin was used as a loading control. (TIF) [file pone.0130340.s008.tif]
